# The mediated effect of HIV risk perception in the relationship between peer education and HIV testing uptake among three key populations in China

**DOI:** 10.1186/s12981-021-00334-2

**Published:** 2021-03-25

**Authors:** Yuxi Lin, Chuanxi Li, Lin Wang, Kedi Jiao, Wei Ma

**Affiliations:** Department of Epidemiology, School of Public Health, Cheeloo College of Medicine, Shandong University, 44 West Wenhua Road, Jinan, Shandong 250012 People’s Republic of China

**Keywords:** HIV testing, Peer education, HIV risk perception, Mediated effect, Key populations

## Abstract

**Background:**

Peer education and HIV risk perception are related to HIV testing uptake among key populations. We aimed to examine the association between peer education, HIV risk perception, and HIV testing uptake, as well as to evaluate the mediated effect of HIV risk perception in the relationship between peer education and HIV testing uptake.

**Methods:**

We conducted a cross-sectional survey among 1188 HIV-uninfected or unknown participants from populations of men who have sex with men (MSM), female sex workers (FSWs), and drug users (DUs) in seven cities of China. Partial correlation analysis and regression analysis were employed to examine the associations among peer education, HIV risk perception, and HIV testing uptake. Mediation analysis was conducted to assess whether HIV risk perception mediated the hypothesized association.

**Results:**

Receiving peer education was associated with higher odds of HIV testing uptake among MSM, FSWs and DUs. Perceiving risk of HIV infection was associated with higher odds of HIV testing uptake among MSM and DUs. Among MSM, the relationship between peer education and HIV testing uptake was mediated by moderate risk perception of HIV (indirect effect: 0.53, 95% CI 0.07 to 1.21), and by high risk perception of HIV (indirect effect: 0.50, 95% CI 0.01 to 1.17). Among DUs, the relationship between peer education and HIV testing uptake was mediated by moderate risk perception of HIV (indirect effect: 1.80, 95% CI 0.57 to 3.45).

**Conclusions:**

Participants who received peer education tended to perceive their risk of HIV infection, which in turn was associated with increased HIV testing uptake among MSM and DUs. Therefore, in addition to peer education interventions, more report about HIV epidemic and risk assessment should also be scaled up to enhance HIV risk perception among key populations.

## Background

In China, sexual transmission is the current primary route of HIV transmission [1]. Among the new diagnosed HIV/AIDS cases in 2019, sexual transmission accounted for 96.7% (male–male sexual transmitted cases accounted for 23.0%, and heterosexual transmitted cases accounted for 73.7%) [2]. Men who have sex with men (MSM), female sex workers (FSWs), and drug users (DUs) are key populations for HIV infection in China due to their unsafe sexual behaviors, such as unprotected sex and multiple sexual partners [3–5].

HIV testing is an essential step for effective HIV prevention [6], offering the possibility to receive treatment, change sexual behavior and improve health and quality of life [7]. The first goal of the 90-90-90 cascade target proposed by the Joint United Nations Programme on HIV/AIDS (UNAIDS) is that 90% of people living with HIV know their HIV status by 2020 [8]. At present, this goal have only been achieved within limited regions [9, 10], and HIV testing services are still underutilized among key populations around the world, given that multiple factors such as poor HIV-related knowledge, stigma, and discrimination hinder their access to HIV services [11–13]. Globally, about half of key populations living with HIV are not aware of their HIV status [14]. In China, the annual HIV testing rate among MSM is low, ranging from 44.6 to 69% [15], followed by DUs with an annual HIV testing proportion of 25.8% [16] and FSWs of 24.4% [5]. It is therefore imperative to promote HIV testing uptake among key populations to control HIV epidemic.

Peer education intervention is a widely used strategy targeting key populations to promote HIV prevention and treatment, involving recruiting members of a specific group at-risk for HIV to encourage peers to improve HIV knowledge and reduce risky sexual behaviors [17–19]. A previous meta-analysis proved that peer education can effectively promote HIV testing uptake among key populations [18], and another systematic review also demonstrated that the information about the location, availability and the importance of HIV testing services for key populations provided by peer education are related to HIV testing uptake [20]. A peer-based intervention program conducted among MSM in China has proved that peer education can be used to improve HIV testing and linkage to care [21]. Additionally, an intervention model in Vietnam has been proved effective to improve HIV risk perception among community and factory youth [19].

To date, only about half of key populations perceived their risk of HIV infection globally [22, 23]. The relationship between risk perception and HIV testing uptake among key populations have been evaluated in a variety of studies, however, the results are conflicting. For example, a cross-section study conducted among MSM in China indicated that those who perceived higher risk of HIV tended to test for HIV [22], and a study conducted among DUs indicated that perceptions of no risk or low-risk for HIV infection was barrier to HIV testing services [24]. Besides, some studies conducted in sub-Saharan Africa, Germany and India also demonstrated that risk perception for HIV is one of facilitators of HIV testing uptake among MSM, FSWs and DUs, because they might want to know their results when perceived their risk of HIV infection [20, 25, 26]. On the contrary, a study conducted in Peru showed that those participants who perceived high risk for HIV were less likely to test frequently [27].

There is a dearth of studies exploring the mediated effect of HIV risk perception in the relationship between peer education and HIV testing uptake. Previous studies assessed the relationship among peer education, risk perception of HIV and HIV testing uptake separately. In this study, we examined these relationships among three key populations in China. Furthermore, we explored whether risk perception of HIV mediated the relationship between peer education and HIV testing uptake. We hypothesized that (1) peer education is positively associated with HIV testing uptake among MSM, FSWs, and DUs and (2) the association is mediated through the risk perception of HIV infection.

## Methods

### Participants

A cross-sectional survey was conducted among three groups at-risk for HIV in seven cities of China in 2018, with MSM recruited from Shijiazhuang and Xiamen, FSWs recruited from Zhengzhou, Nanchang and Biyang, and DUs recruited from Qingdao and Shanghai. Considering the capacity for recruiting adequate key populations and the feasibility of the survey, we selected two provinces from the north and south of China for each key population. Considering the speed of recruitment, we chose the capital of targeting provinces as study sites for different populations, and added Biyang as one of study sites for FSWs.

Convenience sampling was used to recruit participants with the help of local community based organizations (CBOs). Prior to beginning this survey, all people accessing to CBOs were confirmed their eligibility and those eligible person were invited to participate in this survey by staff of local CBOs. Those provided informed consent and voluntarily participated in this study were included in this survey. The inclusion criteria included: (1) 18 years or older; (2) had high-risk sexual behaviors (had sex with men for MSM, exchanged sex for money or other goods for FSWs, and took drugs for DUs) in the past 12 months; (3) self-reported HIV infection status was negative or unknown; and (4) provided informed consent and voluntarily participated in this study.

### Procedures

Those who were willing to participate in study would be directed in a quiet and private room in local CBOs. The purpose, content and procedures of this study were explained to the eligible participants by a trained team member and signed informed consent was obtained. Every participant was assigned a code to obtain questionnaires. Staff then oriented participants to input their codes through smartphones to get the electronic questionnaires. Paper questionnaires were also prepared for those who did not use smartphones. Participants who completed the questionnaires were reimbursed 50 RMB (7.55 USD).

### Measures

#### Social-demographics

Participants were asked about their gender, age, time of residence, education level, monthly income and marital status. MSM also reported their sexual orientation and DUs reported the type of drugs they used and the route of drug use.

#### Sexual behaviors

We measured participants’ high-risk sexual behaviors by asking about whether or not they used condom consistently and experienced condom breakage or slippage in anal sex in the past month. We dichotomized these variable after removing those who did not had anal sex in the past month.

MSM participants answered 4 questions about their condom use frequency during sex with their male fixed sexual partners, non-commercial male casual sexual partners, commercial male sexual partners and female sexual partners in the past month. FSWs participants answered 2 questions about their condom use frequency during sex with their clients in the past month and with their husband/boyfriend in the past month. DUs participants answered 4 questions about their condom use frequency during sex with partners after using drugs, in multiple-partner sexual behaviors, in sex with spouse, and with commercial sexual partners in the past month. In the analysis, we dichotomized this variable (“Never”, “Sometimes”, or “Every time”) into “had condomless sex” and “did not have condomless sex”. Participants who reported “Never” or “Sometimes” were categorized as had condomless sex in the past month.

MSM participants were asked if they had experienced condom breakage or slippage in anal sex with male sexual partners in the past month. FSWs participants were asked if they had experienced condom breakage or slippage in sex with their clients in the past month. DUs participants were asked if they had experienced condom breakage or slippage during sexual intercourse with partners after using drugs in the past month. Participants who answered “Yes” were categorized as “experienced condom breakage of slippage in sex in the past month”.

#### Peer education

Participants was asked if they had participated peer education concerning HIV prevention in the past year (“Yes” or “No”). Those responded “Yes” were categorized as “having received peer education”. Peer education was defined as activity in which peer educators from key populations deliver the knowledge about HIV prevention or information about HIV related services through multiple methods, including face-to-face conversation, posters, leaflets, lectures or internet.

#### Risk perception of HIV infection

Participants were asked if they felt that the HIV infection among MSM/FSWs/DUs is serious (“Serious”, “Moderate”, “Low”, or “Don’t know”). Those responded “Serious” were categorized as “perceived high risk of HIV infection”, those responded “Moderate” were categorized as “perceived moderate risk of HIV infection”, and those responded “Low” or “Don’t know” were categorized as “did not perceive risk of HIV infection”.

#### History of HIV testing

Participants were asked about their HIV testing history (“Never tested”, “Tested before 12 months”, “Tested one time in the past 12 months”, or “Tested two times or more in the past 12 months”). In analyses, we dichotomized this variable into had versus did not have testing for HIV in the past 12 months. Those who responded “Tested one time in the past 12 months” or “Tested two times or more in the past 12 months” were categorized had HIV testing in the past 12 months.

### Statistical analysis

The database was developed by one team member, with the data of electric questionnaires exported from “Wenjuanxing”, a professional platform for online questionnaires distribution, filling and collection, and the data of paper questionnaires inputted by EpiData 3.1. A total of 1220 questionnaires was received, and 32 questionnaires were excluded due to responses with missing data, leaving a finial sample size of 1188 participants. Descriptive analysis and partial correlation analysis were conducted in SPSS 24.0 and mediation analysis was conducted in the software R4.0.2. All statistic tests were two-sided, and *p* < 0.05 was considered as statistically significant. We conducted chi-square tests to assess the difference of the distribution of each variable among groups of participants categorized by their history of HIV testing in the past 12 months.

To assess the conditional effects of variables on HIV testing uptake, we fitted 3 separate multivariable logistic regression models. The first model assessed socio-demographic correlates of HIV testing uptake, variables with a *p*-value higher than 0.3 were excluded in subsequent models [28]. Then the second model was fitted to assess the conditional effects of high-risk sexual behavioral associated with HIV testing uptake after removing those who did not have sex in the past month. The third model was fitted to assess peer education and risk perception of HIV associated with HIV testing uptake.

Then, we examine the partial correlations between peer education, risk perception of HIV and HIV testing uptake among MSM, FSWs, and DUs.

**Fig. 1:**
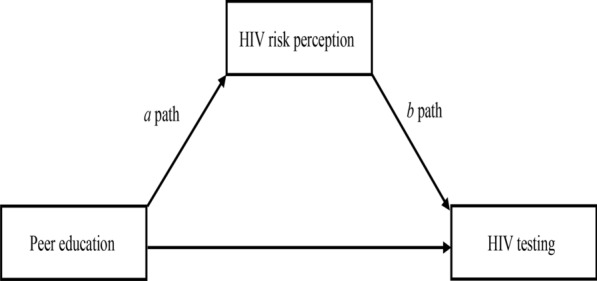
The *a* path and *b* path in mediation analysis

Meditation analyses were conducted among every different populations. First, we conducted multinomial logistic regression for different levels of HIV risk perception on peer education; doing so provided us with the “*a*” path (Fig. 1) parameter estimates and standard errors. Then, we fitted a logistic regression model for HIV testing uptake on peer education and HIV risk perception, and used the parameter estimates and standard errors of moderate and high HIV risk perception as our “*b*” path (Fig. 1). Finally, we used the “medci” function of “RMediation” package in the software R4.0.2 to estimate each mediated effect and its asymmetric confidence interval (CI) to determine whether mediated effects were significant [29]. The alpha was 0.05, and type was “prodclin”. A significant mediation effect existed when CI did not contain zero.

## Results

Descriptive statistics are presented in Table 1. We received a total of 1227 questionnaires, 39 incomplete questionnaires were excluded. A total of 1188 participants were included in this study, including 419 MSM, 400 FSWs and 369 DUs. About one third (127/419, 30.3%) MSM, one quarter (93/400, 23.3%) FSWs and 44.4% (164/369) DUs reported that they had not been tested for HIV in the past 12 months. Participants who had HIV testing in the past 12 months had a higher proportion of receiving peer education and perceiving moderate or high HIV risk among three populations (*p* < 0.05) (Table 1).

**Table 1:** Socio-demographic, sexual behavioral, peer education, and HIV risk perception characteristics comparing participants who had HIV testing to those who had not in the past 12 months among three key populations in China (N = 1188)

Variables	HIV testing in the past 12 months								
	MSM (N = 419)			FSWs (N = 400)			DUs (N = 369)		
	No n (%)	Yes n (%)	*p*	No n (%)	Yes n (%)	*p*	No n (%)	Yes n (%)	*p*
Gender									
Male	127 (100.0)	292 (100.0)	–	–	–		75 (45.7)	148 (72.2)	** < 0.001**
Female	–	–		93 (100.0)	307 (100.0)		89 (54.3)	57 (27.8)	
Age									
18–25	72 (56.7)	154 (52.7)	0.690	18 (19.4)	19 (6.2)	** < 0.001**	20 (12.2)	84 (41.0)	** < 0.001**
26–35	29 (22.8)	79 (27.1)		36 (38.7)	87 (28.3)		37 (22.6)	57 (27.8)	
36–45	14 (11.0)	37 (12.7)		15 (16.1)	146 (47.6)		54 (32.9)	29 (14.1)	
46 or older	12 (9.4)	22 (7.5)		24 (25.8)	55 (17.9)		53 (32.3)	35 (17.1)	
Duration of local residence									
2 years or less	43 (33.9)	81 (27.7)	0.207	49 (52.7)	218 (71.0)	**0.001**	16 (9.8)	32 (15.6)	0.097
More than 2 years	84 (66.1)	211 (72.3)		44 (47.3)	89 (29.0)		148 (90.2)	173 (84.4)	
Education level									
Middle school or lower	23 (18.1)	33 (11.3)	**0.016**	57 (61.3)	250 (81.4)	** < 0.001**	36 (22.0)	50 (24.4)	** < 0.001**
High school	33 (26.0)	54 (18.5)		19 (20.4)	46 (15.0)		86 (52.4)	44 (21.5)	
College or higher	71 (55.9)	205 (70.2)		17 (18.3)	11 (3.6)		42 (25.6)	111 (54.1)	
Monthly income (RMB)^a^									
3000 or less	57 (44.9)	115 (39.4)	0.483	50 (53.8)	227 (73.9)	**0.001**	93 (56.7)	96 (46.8)	**0.021**
3001–5000	37 (29.1)	86 (29.5)		26 (28.0)	50 (16.3)		38 (23.2)	75 (36.6)	
5001 or higher	33 (26.0)	91 (31.2)		17 (18.3)	30 (9.8)		33 (20.1)	34 (16.6)	
Marital status									
Single	95 (74.8)	227 (77.7)	0.626^b^	30 (32.3)	51 (16.6)	**0.004**	75 (45.7)	161 (78.5)	** < 0.001**
Married/co-habitation	28 (22.0)	53 (18.2)		53 (57.0)	208 (67.8)		72 (43.9)	38 (18.5)	
Divorced/widowed/segregated	4 (3.1)	12 (4.1)		10 (10.8)	48 (15.6)		17 (10.4)	6 (2.9)	
Sexual orientation									
Gay	57 (44.9)	203 (69.5)	** < 0.001**						
Other	70 (55.1)	89 (30.5)							
Types of drugs used									
Single							119 (72.6)	146 (71.2)	0.776
Multiple							45 (27.4)	59 (28.8)	
Injected drug in the past month									
No							160 (97.6)	193 (94.1)	0.110
Yes							4 (2.4)	12 (5.9)	
Condomless sex (past month)									
No	90 (72.4)	211 (72.3)	0.970	42 (45.2)	149 (48.5)	0.568	106 (64.6)	89 (43.4)	** < 0.001**
Yes	35 (27.6)	81 (27.7)		51 (54.8)	158 (51.5)		58 (35.4)	116 (56.6)	
Condom breakage or slippage (past month)									
No	115 (90.6)	265 (90.8)	0.948	38 (40.9)	123 (40.1)	0.891	109 (66.5)	133 (64.9)	0.750
Yes	12 (9.4)	27 (9.2)		55 (59.1)	184 (59.9)		55 (33.5)	72 (35.1)	
Peer education									
No	86 (67.7)	135 (46.2)	** < 0.001**	62 (66.7)	96 (31.3)	** < 0.001**	55 (33.5)	25 (12.2)	** < 0.001**
Yes	41 (32.3)	157 (53.8)		31 (33.3)	211 (68.7)		109 (66.5)	180 (87.8)	
HIV risk perception									
No	65 (51.2)	88 (30.1)	** < 0.001**	67 (72.0)	189 (61.6)	**0.005**	125 (76.2)	69 (33.7)	** < 0.001**
Moderate	31 (24.4)	92 (31.5)		18 (19.4)	44 (14.3)		24 (14.6)	88 (42.9)	
High	31 (24.4)	112 (38.4)		8 (8.6)	74 (24.1)		15 (9.1)	48 (23.4)	

Bolded *p* values are statistically significant at *p* < 0.05

^a^1 USD = 6.62 RMB in 2018

^b^Fisher’s exact test

Tables 2, 3 and 4 present factors independently associated with HIV testing uptake among MSM, FSWs and DUs, respectively. Table 2 shows that sexual orientation, education level, peer education and HIV risk perception were related to HIV testing uptake in the past 12 months among MSM (*p* < 0.05). Table 3 shows that age, duration of local residence, education level and peer education were related to HIV testing among FSWs (*p* < 0.05). Table 4 shows that gender, age, education level, monthly income, marital status, peer education and HIV risk perception were related to HIV testing uptake among DUs (*p* < 0.01).

**Table 2:** Factors associated with HIV testing in the past 12 months among MSM in China

Variables	Model 1 (N = 419)		Model 2^a^ (N = 260)		Model 3 (N = 419)	
	AOR (95%CI)	*p* value	AOR (95%CI)	*p* value	AOR (95%CI)	*p* value
Age						
18–25	Ref	–				
26–35	1.32 (0.72, 2.42)	0.373				
36–45	1.34 (0.56, 3.21)	0.509				
46 or older	1.30 (0.45, 3.76)	0.631				
Duration of local residence						
2 years and less	Ref	–				
More than 2 years	1.15 (0.70, 1.89)	0.583				
Education						
Middle school or lower	Ref	–	Ref	**–**	Ref	–
High school	1.01 (0.49, 2.08)	0.974	1.29 (0.50, 3.32)	0.605	1.25 (0.59, 2.63)	0.566
College or higher	1.87 (0.97, 3.58)	0.061	1.63 (0.73, 3.66)	0.232	**2.24 (1.17, 4.29)**	**0.015**
Monthly income (RMB)^b^						
3000 or less	Ref	–				
3001–5000	1.20 (0.68, 2.10)	0.534				
5001 or higher	1.25 (0.68, 2.28)	0.476				
Marital status						
Single	Ref	–				
Married/co-habitation	0.91 (0.43, 1.20)	0.800				
Divorced/widowed/segregated	1.00 (0.28, 3.58)	0.999				
Sex orientation						
Gay	Ref	–	Ref	–	Ref	–
Other	**0.36 (0.23, 0.56)**	** < 0.001**	**0.49 (0.28, 0.88)**	**0.018**	**0.38 (0.24, 0.60)**	** < 0.001**
Condomless sex (past month)						
No			Ref	–		
Yes			0.65 (0.36, 1.18)	0.155		
Condom breakage or slippage (past month)						
No			Ref	–		
Yes			0.72 (0.30, 1.73)	0.466		
Peer education						
No					Ref	–
Yes					**2.20 (1.38, 3.50)**	**0.001**
HIV risk perception						
No					Ref	–
Moderate					**2.00 (1.15, 3.47)**	**0.014**
High					**2.79 (1.62, 4.81)**	** < 0.001**

Bolded *p* values are statistically significant at *p* < 0.05

^a^Excluded those had not anal sex in the past month

^b^1 USD = 6.62 RMB in 2018

**Table 3:** Factors associated with HIV testing in the past 12 months among FSWs in China

Variables	Model 1 (N = 400)		Model 2^a^ (N = 398)		Model 3 (N = 400)	
	AOR (95%CI)	*p* value	AOR (95%CI)	*p* value	AOR (95%CI)	*p* value
Age						
18–25	Ref	–	Ref	–	Ref	–
26–35	1.55 (0.62, 3.87)	0.346	1.39 (0.55, 3.53)	0.483	1.33 (0.50, 3.54)	0.570
36–45	**4.51 (1.65, 12.31)**	**0.003**	**3.88 (1.39, 10.82)**	**0.010**	**2.95 (1.00, 8.67)**	**0.049**
46 or older	0.95 (0.34, 2.68)	0.922	0.80 (0.28, 2.30)	0.672	0.66 (0.21, 2.02)	0.461
Duration of local residence						
2 years and less	Ref	–	Ref	–	Ref	–
More than 2 years	**0.56 (0.32, 0.95)**	**0.032**	**0.56 (0.33, 0.96)**	**0.034**	0.75 (0.42, 1.32)	0.320
Education						
Middle school or lower	Ref	–	Ref	**–**	Ref	**–**
High school	0.63 (0.32, 1.25)	0.183	0.63 (0.31, 1.25)	0.184	0.51 (0.25, 1.06)	0.071
College or higher	**0.24 (0.10, 0.58)**	**0.001**	**0.22 (0.09, 0.55)**	**0.001**	**0.19 (0.07, 0.49)**	**0.001**
Monthly income (RMB)^b^						
3,000 or less	Ref	–	Ref	–	Ref	–
3001–5000	0.63 (0.34, 1.18)	0.152	0.61 (0.32, 1.15)	0.126	0.66 (0.34, 1.29)	0.224
5001 or higher	0.73 (0.33, 1.58)	0.416	0.71 (0.33, 1.56)	0.397	0.86 (0.39, 1.91)	0.707
Marital status						
Single	Ref	–	Ref	–	Ref	–
Married/co-habitation	1.87 (0.92, 3.80)	0.086	1.96 (0.94, 4.10)	0.074	1.81 (0.82, 4.00)	0.141
Divorced/widowed /Segregated	2.13 (0.80, 5.65)	0.130	2.25 (0.84, 6.05)	0.109	1.95 (0.68, 5.62)	0.217
Condomless sex (past month)						
No			Ref	–		
Yes			0.76 (0.44, 1.33)	0.336		
Condom breakage or slippage (past month)						
No			Ref	–		
Yes			0.90 (0.52, 1.55)	0.701		
Peer education						
No					Ref	–
Yes					**3.51 (1.94, 6.33)**	** < 0.001**
HIV risk perception						
No					Ref	–
Moderate					0.63 (0.29, 1.35)	0.234
High					1.80 (0.74, 4.38)	0.198

Bolded *p* values are statistically significant at *p* < 0.05

^a^Excluded those had not anal sex in the past month

^b^1 USD = 6.62 RMB in 2018

**Table 4:** Factors associated with HIV testing in the past 12 months among DUs in China

Variables	Model 1 (N = 369)		Model 2^a^ (N = 210)		Model 3 (N = 369)	
	AOR (95%CI)	*p* value	AOR (95%CI)	*p* value	AOR (95%CI)	*p* value
Gender						
Male	Ref	–	Ref	–	Ref	–
Female	**0.28 (0.16, 0.49)**	** < 0.001**	**0.18 (0.08, 0.45)**	** < 0.001**	**0.36 (0.18, 0.69)**	**0.002**
Age						
18–25	Ref	–	Ref	–	Ref	–
26–35	0.52 (0.23, 1.17)	0.112	0.71 (0.24, 2.10)	0.539	**0.18 (0.06, 0.52)**	**0.002**
36–45	**0.24 (0.10, 0.60)**	**0.002**	0.41 (0.09, 1.85)	0.243	**0.09 (0.03, 0.29)**	** < 0.001**
46 or older	**0.18 (0.07, 0.46)**	** < 0.001**	0.19 (0.04, 0.99)	0.048	**0.05 (0.02, 0.17)**	** < 0.001**
Duration of local residence						
2 years and less	Ref	–				
More than 2 years	1.22 (0.54,2.79)	0.635				
Education						
Middle school or lower	Ref	–	Ref	–	Ref	–
High school	**0.11 (0.05, 0.25)**	** < 0.001**	0.40 (0.10, 1.66)	0.206	**0.12 (0.05, 0.28)**	** < 0.001**
College or higher	**0.31 (0.13, 0.75)**	**0.009**	0.72 (0.16, 3.27)	0.668	0.62 (0.23, 1.70)	0.358
Monthly income (RMB)^b^						
3,000 or less	Ref	–	Ref	–	Ref	–
3001–5000	**5.06 (2.41, 10.62)**	** < 0.001**	**8.36 (2.57, 27.18)**	** < 0.001**	**4.72 (2.07, 10.78)**	** < 0.001**
5,001 or higher	1.63 (0.75, 3.51)	0.216	**4.08 (1.22, 13.58)**	**0.022**	1.32 (0.57, 3.08)	0.518
Marital status						
Single	Ref	–	Ref	–	Ref	–
Married/Co-habitation	**0.14 (0.07, 0.28)**	** < 0.001**	**0.11 (0.03, 0.33)**	** < 0.001**	**0.24 (0.11, 0.52)**	** < 0.001**
Divorced/Widowed /Segregated	**0.18 (0.05, 0.58)**	**0.004**	**0.11 (0.02, 0.61)**	**0.011**	**0.22 (0.06, 0.85)**	**0.028**
Types of drugs used						
Single	Ref	–				
Multiple	1.08 (0.60, 1.96)	0.790				
Injected drug in the past month						
No	Ref	–				
Yes	0.84 (0.22, 3.27)	0.800				
Condomless sex (past month)						
No			Ref	–		
Yes			1.09 (0.43, 2.75)	0.856		
Condom breakage or slippage (past month)						
No			Ref	–		
Yes			0.52 (0.24, 1.12)	0.094		
Peer education						
No					Ref	–
Yes					**16.93 (6.12, 4 6.82)**	** < 0.001**
HIV risk perception						
No					Ref	–
Moderate					**4.23 (2.03, 8.83)**	** < 0.001**
High					**4.21 (1.81, 9.77)**	**0.001**

Bolded *p* values are statistically significant at *p* < 0.05

^a^Excluded those had not anal sex in the past month

^b^1 USD = 6.62 RMB in 2018

Partial correlations among peer education, risk perception of HIV and HIV testing uptake in the past 12 months among MSM, FSWs and DUs are shown in Table 5. Among MSM and DUs, peer education, risk perception of HIV and HIV testing uptake were interrelated with each other among every population (*p* < 0.001). Among FSWs, peer education was related to HIV risk perception and HIV testing uptake (*p* < 0.001), however, the correlation of HIV risk perception and HIV testing uptake was not statistically significant (*p* > 0.05).

**Table 5:** Partial correlations between peer education, HIV risk perception infection and HIV testing among MSM, FSWs, and DUs in China

Variable	Group	HIV risk perception		HIV testing		HIV risk perception → HIV testing	
		r	*p* value	r	*p* value	r	*p* value
Peer education	MSM^a^	0.10	**0.044**	0.19	** < 0.001**	0.20	** < 0.001**
	FSWs^b^	0.27	** < 0.001**	0.28	** < 0.001**	0.09	0.073
	DUs^c^	0.15	**0.004**	0.32	** < 0.001**	0.34	** < 0.001**

Bolded *p* values are statistically significant at *p* < 0.05

^a^The results adjusted for education level and sexual orientation

^b^The results adjusted for age, duration of local residence and education level

^c^The results adjusted for gender, age, education level, monthly income and married status

Table 6 presents *a* paths, *b* paths, estimates and 95% CIs for mediated effects among MSM, FSWs and DUs. Among MSM, the mediated effects of moderate and high HIV risk perception (indirect effect: 0.53, 95% CI 0.07, 1.21; and indirect effect: 0.50, 95% CI 0.01, 1.17) in the relationship between peer education and HIV testing were significant when compared to perceived of no HIV risk. Besides, among DUs, compared to not perceived the risk of HIV, only moderate risk perception of HIV mediated (indirect effect: 1.80, 95% CI 0.57, 3.45) the relationship of peer education to HIV testing. However, the mediated effects of moderate and high risk perception of HIV were not statistically significant (indirect effect: −0.52, 95% CI −1.44, 0.33; and indirect effect: 0.76, 95% CI −0.61, 2.52) in the relationship of peer education on HIV testing uptake among FSWs.

**Table 6:** Mediation analysis of HIV risk perception in the relationship between peer education and HIV testing among overall three key populations, among MSM, FSWs, and DUs in China

Group	Level of risk perception of HIV	*a* path (peer education → HIV risk perception)	*b* path (HIV risk perception → HIV testing)	Mediated effect	
		Est (se)	Est (se)	Est (se)	95% CI
MSM^a^	Moderate	0.76 (0.25)**	0.69 (0.28)*	0.53 (0.30)	**[0.07, 1.21]**
	High	0.48 (0.24)*	1.03 (0.28)**	0.50 (0.30)	**[0.01, 1.17]**
FSWs^b^	Moderate	1.13 (0.35)**	−0.50 (0.38)	-0.52 (0.44)	[−1.44, 0.33]
	High	1.60 (0.37)**	0.45 (0.45)	0.76 (0.80)	[−0.61, 2.52]
DUs^c^	Moderate	1.24 (0.34)**	1.44 (0.38)**	1.80 (0.74)	**[0.57, 3.45]**
	High	0.87 (0.49)	1.44 (0.43)**	1.28 (0.87)	[−0.11, 3.27]

^a^The results adjusted for education level and sexual orientation

^b^The results adjusted for age, duration of local residence and education level

^c^The results adjusted for gender, age, education level, monthly income and married status

^*^p < 0.05; **p < 0.01

Bolded CI does not contain zero

## Discussion

In this survey conducted among MSM, FSWs and DUs in 7 cities in China, we found that receiving peer education was positively associated with HIV risk perception and HIV testing uptake among three key populations, and that HIV risk perception was positively associated with HIV testing uptake among MSM and DUs. We also found that HIV risk perception mediated the relationship between peer education and HIV testing uptake among MSM and DUs. This study extends previous research on relationship between peer education, HIV risk perception and HIV testing uptake separately by exploring the mediated effect of HIV risk perception in the relationship between peer education and HIV testing uptake.

The results showed that peer education was positively associated with HIV testing uptake among MSM, FSWs and DUs. This finding confirmed and added to previous meta-analysis that demonstrated peer education had a positive impact on promoting HIV testing uptake among MSM, IDUs and FSWs [18]. Firstly, peer education could advance the awareness of the importance of HIV testing and provide the information about the location and availability of HIV services, which increase accessing to HIV testing services [20]. Second, peers could offering social support and referrals for services, which improve HIV testing uptake [30]. Besides, the emotional support provided by peers could help key populations overcome specific barriers to accessing HIV testing services [31]. Additionally, compared to traditional interventions, peer education intervention is more trustable and confidential for key populations, thus can better reach “hidden” populations who may have limited access to traditional outreach [21, 32]. Moreover, the cost-effect of peer education is also less than normal outreach [33, 34]. Therefore, peer education about HIV testing should be strengthened and scaled up to improve HIV testing uptake among key populations.

In this study, compared to perception of no HIV risk, perception of moderate and high HIV risk not only directly affected HIV testing uptake, but also mediated the effects of peer education on HIV testing uptake among MSM. Among group of DUs, moderate perception of HIV risk mediated the relationship between peer education and HIV testing uptake. Those participants who have received peer education about HIV prevention tended to perceive their risk of HIV infection, and in turn were more likely to be tested for HIV. This finding is consistent with the health belief model, which shows that knowledge is the premise of risk perception and when people perceiving a risk for a health threat, including perceived susceptibility and perceived severity of the threat, they will be more likely to participate in changing health related behaviors [35]. There is a dearth of studies exploring the mediated effect of HIV risk perception in the association between peer education and HIV testing, but the relationship of HIV risk perception to peer education and to HIV testing have been widely documented. Our finding is in line with the previous study, indicating that peer education had positive association with HIV risk perception [19] and with study showed that HIV risk perception has positive association with HIV testing uptake among MSM [25]. Therefore, in addition to peer education, strategies improving HIV risk perception are necessary to advance HIV testing uptake among key populations. The report about the HIV epidemic and education on HIV-related knowledge among key populations should be scaled up to improve awareness of HIV risk. Besides, prevention providers should strengthen their efforts in risk assessment and recommend people with high risk of HIV infection for testing.

We found that peer education had an effect on moderate risk perception of HIV, but the effect on high perception of HIV risk was not statistically significant among DUs when compared to no perception of HIV risk. It is likely that some DUs underestimated their risk of HIV infection. A study showed that the use of drugs increased the risk of HIV transmission by encouraging high-risk sexual behaviors, such as condomless sex and multiple sexual partners, which were related to increasing HIV infection risk [36]. In our study, most of the participants did not inject drug in the past month, so they might perceive that they had no or low risk of HIV infection because they did not sharing needles and ignored their risk of HIV infection through sexual transmission. Previous study showed that not sharing needles might be a reason of underestimated their risk of HIV infection among DUs [37]. More education on HIV transmission and risk factors of HIV infection are needed to help DUs to accurately assess their risk for acquiring HIV.

It is notably that there is a possible mediated effect of HIV risk perception among FSWs, however, this effect is not statistically significant. Further large-scale studies are needed to confirm this finding. We found that peer education was associated with risk perception of HIV, while perception of HIV infection was not associated with HIV testing when adjusting peer education. These findings suggest that FSWs as an illegal position in China suffered multiple barriers to access to HIV testing services though they had perceived their HIV risk. Factors hindering accessing to HIV testing services frequently investigated among FSWs are, poor HIV-related knowledge, stigma, and discrimination [12, 38–40]. In order to increase HIV testing uptake, in addition to peer education and HIV risk perception interventions, further research should explore effective interventions to help FSWs overcoming these difficulties to accessing HIV testing services.

There are limitations to this study. Firstly, limited by the nature of cross-sectional study, the temporal relationship between variables were not clear, and therefore causal relationships could not be warranted. Specifically, the main variables were measured in different time scales, peer education and history of HIV testing was measured within the past 12 months, while the risk perception was measured when the survey was conducted. Secondly, study participants were recruited at CBOs, and therefore may not have been representative of key populations who were not accessing these sites. Thirdly, we used only a single-item to measure HIV risk perception. Further study are suggested to use a comprehensive measure to evaluate HIV risk perception. Finally, because our participants were key populations, their responses were subject to social desirability, thus some response might be inaccurate, especially those related to sexual behaviors and HIV. However, most interviews were administered online, which would enhance the reliability of data to some extent.

## Conclusion

In summary, our study demonstrated that the risk perception of HIV was an important mediator of peer education and HIV testing uptake among key populations, especially among MSM and DUs. Therefore, in addition to peer education interventions, more report about HIV epidemic and risk assessment should also be strengthen to enhance HIV risk perception among key populations.

## Data Availability

The datasets analyzed during the current study are not publicly available but are available from the corresponding author on reasonable request.

## Abbreviations

HIV: Human immunodeficiency virus
AIDS: Acquired immune deficiency syndrome
CBOs: Community-based organizations
MSM: Men who have sex with men
FSWs: Female sex workers
DUs: Drug users
UNAIDS: The Joint United Nations Programme on HIV/AIDS
